# Methamphetamine Activates Trace Amine Associated Receptor 1 to Regulate Astrocyte Excitatory Amino Acid Transporter-2 via Differential CREB Phosphorylation During HIV-Associated Neurocognitive Disorders

**DOI:** 10.3389/fneur.2020.593146

**Published:** 2020-11-25

**Authors:** Irma E. Cisneros, Anuja Ghorpade, Kathleen Borgmann

**Affiliations:** Department of Microbiology, Immunology, and Genetics, University of North Texas Health Science Center, Fort Worth, TX, United States

**Keywords:** calcium, cyclic AMP, excitotoxicity, glutamate, inflammation, kinase activation

## Abstract

Methamphetamine (METH) use, referred to as methamphetamine use disorder (MUD), results in neurocognitive decline, a characteristic shared with HIV-associated neurocognitive disorders (HAND). MUD exacerbates HAND partly through glutamate dysregulation. Astrocyte excitatory amino acid transporter (EAAT)-2 is responsible for >90% of glutamate uptake from the synaptic environment and is significantly decreased with METH and HIV-1. Our previous work demonstrated astrocyte trace amine associated receptor (TAAR) 1 to be involved in EAAT-2 regulation. Astrocyte EAAT-2 is regulated at the transcriptional level by cAMP responsive element binding (CREB) protein and NF-κB, transcription factors activated by cAMP, calcium and IL-1β. Second messengers, cAMP and calcium, are triggered by TAAR1 activation, which is upregulated by IL-1β METH-mediated increases in these second messengers and signal transduction pathways have not been shown to directly decrease astrocyte EAAT-2. We propose CREB activation serves as a master regulator of EAAT-2 transcription, downstream of METH-induced TAAR1 activation. To investigate the temporal order of events culminating in CREB activation, genetically encoded calcium indicators, GCaMP6s, were used to visualize METH-induced calcium signaling in primary human astrocytes. RNA interference and pharmacological inhibitors targeting or blocking cAMP-dependent protein kinase A and calcium/calmodulin kinase II confirmed METH-induced regulation of EAAT-2 and resultant glutamate clearance. Furthermore, we investigated METH-mediated CREB phosphorylation at both serine 133 and 142, the co-activator and co-repressor forms, respectively. Overall, this work revealed METH-induced differential CREB phosphorylation is a critical regulator for EAAT-2 function and may thus serve as a mechanistic target for the attenuation of METH-induced excitotoxicity in the context of HAND.

## Introduction

Methamphetamine use disorder (MUD) is correlated to heightened transmission of human immunodeficiency virus (HIV) (1–3) and increases the severity and onset of HIV-associated neurocognitive disorders (HAND) (4, 5). Cognitive decline observed in the HIV+, methamphetamine (METH) using population is partly attributed to glutamate dysregulation (2, 6, 7). As a major excitatory neurotransmitter in the CNS, optimal glutamate concentrations are vital for learning, memory, problem solving and comprehension. Glutamate imbalances are linked to mental disorders including autism, schizophrenia and depression (8, 9). Glutamate homeostasis and uptake from the synaptic cleft is primarily mediated via excitatory amino acid transporters (EAATs) [as reviewed in (10, 11)]. Of the five human glutamate transporters, EAAT-2 is predominantly expressed by astrocytes and is responsible for >90% of total glutamate uptake in the brain (10, 12, 13). *In vitro*, HIV-1 results in EAAT-2 downregulation and reduced glutamate uptake in astrocytes (14–16). Glutamate, partially mediates the toxic outcomes of METH, inducing excitotoxicity (17–19). However, direct mechanisms of METH-induced EAAT-2 downregulation in astrocytes remain unclear. We previously demonstrated decreased astrocyte EAAT-2 and impaired glutamate uptake following METH and HIV-1 exposure (7). Furthermore, we identified astrocyte trace amine associated receptor (TAAR) 1 is activated by METH, leading to increased intracellular cAMP and regulation of astrocyte EAAT-2 (7). Here, we investigate signal transduction cascades, downstream of TAAR1 regulation and activation, to elucidate the mechanisms of astrocyte EAAT-2 downregulation.

We have previously shown METH-induced EAAT-2 downregulation may be partially due to the activation of astrocyte TAAR1 (7). Of the six functional human TAAR genes, TAAR1 is reported in multiple organs and within several CNS regions, including the prefrontal cortex (20). TAAR1 functions in the neuromodulation of biogenic amines and regulates subcortical monoaminergic transmission and NMDA receptor-mediated glutamate transmission. Thus, TAAR1 plays a critical role in cognitive processing (21–23). TAAR1 activation leads to increased secondary messenger, cAMP, and activation of protein kinase A and C (PKA/C) (24–26). Although the direct mechanism of PKC activation remains vague, PKC becomes activated by increased levels of intracellular calcium [(Ca^+2^)_i_], which is documented to occur following METH exposure (27, 28).

Analysis of the EAAT-2 promoter revealed a cAMP responsive element binding protein (CREB) at −310 and nuclear factor kappa light chain enhancer of activated B cells (NF-κB) elements at −583, −334, −272, and −251 (29, 30), elements associated with immune activation. Dibutyryl cAMP significantly increases EAAT-2 transcription (12), presumably via CREB. For instance, cAMP is well-established to activate transcription of genes containing conserved cAMP responsive elements (CRE)s through the ability to phosphorylate CREB at serine 133 (pCREB^Ser133^). CREB phosphorylation at serine 133 increases dimerization affinity of the CREB binding protein (CBP) and induces transcription. METH increases intracellular cAMP *via* TAAR1 activation in astrocytes (7). NF-κB promoter elements traditionally control transcription of genes involved in the regulation of host immune responses, synaptic plasticity, and memory (31, 32). Previous studies reveal that HIV-1 proteins, including negative regulatory factor (Nef), transactivator of transcription (tat), and glycoprotein 120 (gp120), activate NF-κB signaling pathways in astrocytes (33, 34). Despite METH-induced cAMP increases and HIV-1-mediated NF-κB activation, EAAT-2 expression is reduced by both METH and HIV-1, alone and in combined conditions. While EAAT-2 can be positively or negatively regulated by NF-κB and YY1 (14, 35), the mechanisms dictating CREB-mediated EAAT-2 regulation remains to be investigated. Interestingly, CREB phosphorylation at serine 142, downstream of increased intracellular calcium and calcium/calmodulin kinase (CaMK)II activation, overrides pCREB^Ser133^ transcriptional activation (36, 37).

Glutamate dysregulation is a significant contributing factor to the neurotoxicity associated with METH abuse and HAND. Our previous data demonstrating METH increases intracellular cAMP *via* TAAR1 activation in astrocytes and subsequently regulates EAAT-2 and glutamate clearance levels, sets a strong basis for further investigations of METH-induced TAAR1 signaling in the transcriptional regulation of astrocyte EAAT-2. Therefore, in this study, we investigated the dichotomous outcomes of TAAR1-mediated activation of cAMP/PKA/pCREB^Ser133^ and [Ca^+2^]_i_/CaMKII/pCREB^Ser142^. Our data revealed that differential CREB phosphorylation results in EAAT-2 regulation, indicating that tipping the balance of METH-induced signaling to favor cAMP/PKA/pCREB^Ser133^ serves as a promising countermeasure in reversing METH-induced EAAT-2 downregulation.

## Experimental Procedures

### Isolation, Cultivation, and Activation of Primary Human Astrocytes

Human astrocytes were isolated from first and early second trimester electively aborted specimens as previously described (38, 39). Tissues were procured in full compliance with the ethical guidelines of the National Institutes of Health, Universities of Washington and North Texas Health Science Center. Cell suspensions were centrifuged, washed, suspended in media, and plated at a density of 20 × 10^6^ cells/150 cm^2^. Adherent astrocytes were treated with trypsin and cultured to enhance the purity of replicating astroglial cells. These astrocyte preparations were >99% pure as measured by immunocytochemistry staining for GFAP. Astrocytes were treated with METH [100 or 500 μM, National Institute on Drug abuse (NIDA) Drug Supply Program, Research Resources Identifiers (RRID):SCR_013300], HIV-1_JRFL_ (p24, 10 ng/mL), IL-1β (20 ng/mL, R&D Systems, Minneapolis, MN), N-(3-Ethoxy-phenyl)-4-pyrrolidin-1-yl-23-trifluoromethyl-benzamide (EPPTB, 20 μM, Cat# 4518 Tocris-BioTechne, Minneapolis, MN) (40–43), a cell permeable inhibitor for TAAR1, cAMP-dependent PKA inhibitor, PKI (20 μM, Cat# 476485 Sigma-Aldrich, St. Louis, MO, and Cat# V5681, Promega, Madison, WI) (44–46) and/or CaMKII inhibitor, KN62 (20 μM, Cat# 422706 and I2142, Sigma-Aldrich) (47, 48) at 37°C and 5% CO_2_. HIV-1_JRFL_ was obtained through the NIH AIDS Reagent Program, Division of AIDS, NIAID, NIH: HIV-1 JR-FL Virus from Dr. Irvin Chen (49–51). Normal peripheral blood mononuclear cells (Nebraska Medicine, Apheresis Center, Omaha NE) were isolated and infected with HIV-1_JRFL_ as previously described (52). Culture supernatants were clarified by centrifugation at 10,000 g for 20 min and stored at −80°C. The concentration of HIV-1 JRFL was determined by HIV-1 p24 ELISA (Cat#: XB-1000, Xpress Bio International). Viral stocks were diluted in ASM prior to primary human astrocytes treatment. Astrocytes are not actively infected with HIV-1. Untreated astrocytes were maintained in parallel as control.

### RNA Extraction and Gene Expression Analyses

Astrocyte RNA was isolated 8 h post-treatment, as previously described (53), and mRNA levels were assayed by real-time polymerase chain reaction (PCR). TaqMan 5′ nuclease real-time PCR was performed using StepOnePlus detection system (Thermo Fisher Scientific, Carlsbad, CA). Commercially available TaqMan® Gene Expression Assays were used to measure EAAT-2 (Cat# Hs00188189_m1), TAAR1 (Cat# Hs00373229_s1), PKA (Cat# Hs00427274_m1), CaMKII (Cat# Hs00947041_m1), and glyceraldehyde 3-phosphate dehydrogenase (GAPDH) (Thermo Fisher Scientific; Cat# 4310884E) mRNA levels. GAPDH, a ubiquitously expressed housekeeping gene, was used as an internal normalizing control. The 25 μl reactions were carried out at 48°C for 30 min, 95°C for 10 min, followed by 40 cycles of 95°C for 15 s and 60°C for 1 min in 96-well-optical, real-time PCR plates. Transcripts were quantified by the comparative ΔΔCT method and represented as fold-changes to respective controls.

### Glutamate Clearance Assay

Primary human astrocytes were plated in 48-well-tissue culture plates at a density of 0.15 × 10^6^ cells/well and allowed to recover for 24 h prior to treatment. Following 24 h of treatment, glutamate (400 μM), dissolved in phenol-free astrocyte medium was added into each well, and glutamate clearance was assayed at 10 h post-glutamate addition. The assay was performed and analyzed according to manufacturer's guidelines (Amplex Red Glutamic Acid/Glutamate Oxidase Assay Kit, Cat# A12221, Thermo Fisher Scientific, Carlsbad, CA). Following collection of glutamate supernatants, a colorimetric assay for measurement of metabolic activity was performed using 3-(4,5-dimethylthiazol-2-yl)-2,5-diphenyltetrazolium bromide (MTT, Cat# M2128, Sigma-Aldrich) (54). Briefly, five percent MTT reagent was added to astrocytes and incubated for 20–45 min at 37°C. The MTT solution was removed, and crystals were dissolved in DMSO for 15 min with gentle agitation. Absorbance was assayed at 490 nm in a Spectromax M5 microplate reader (Molecular Devices, Sunnyvale, CA).

### cAMP Assay

Intracellular cAMP levels in astrocytes were measured using a commercially available homogenous, bioluminescence cAMP-Glo™ Assay (Cat# V1502, Promega). Adherent monolayers of astrocytes cultured in 96-well-plates (50,000 cells/well) were stimulated with forskolin (Cat# F686, Sigma-Aldrich) and METH (NIDA Drug Supply Program, RRID:SCR_013300). Cells were activated and lysed in the tissue culture plate. Lysates were diluted to a final cell concentration of ~1,000 cells/μL in lysis buffer supplemented with cAMP specific phosphodiesterase inhibitors [500 μM 3-isobutyl-1-methylxanthine (IBMX, Cat# I7018, Sigma-Aldrich) and 100 μM Ro 20-1724 (Cat# B8279, Sigma-Aldrich)] and transferred to white opaque flat bottom 96-well-assay plates at approximately 5,000 cells/reaction. Intracellular cAMP levels were assayed using GloMax 96 Microplate Luminometer with dual injectors (Promega).

### Kinase Assays

Intracellular PKA and CaMKII levels in astrocytes were measured using a commercially available homogenous, high-throughput screening method for measuring kinase activity, Kinase-Glo® Max Luminescent Assay, (Cat# V6073, Promega) and commercially available PKA Kinase Enzyme System (Cat# V4246, Promega) and CaMKIIα Kinase Enzyme System (Cat# V4018, Promega). cAMP-dependent PKA catalytic subunit α is a 40 kDa bovine recombinant enzyme expressed and purified from *E. coli* (Accession number NM_174584.2). PKA purity was 90% as defined from Promega quality control assays. Human recombinant CaMKIIα was expressed by baculovirus in sf9 insect cells using N-terminal GST tag (Accession number NM_171825, a Ser/Thr protein kinase and a member of the Ca^+2^/calmodulin dependent protein kinase family). The specific activity of CaMKIIα was determined to be 960 nmol/min/mg as per assay protocol. Briefly, adherent monolayers of astrocytes cultured in 96-well-plates (50,000 cells/well) were treated (+/– METH, 500 μM) in PKA or CaMKIIα reaction buffer for 5, 15, or 30 min. Cells were directly activated in the tissue culture plate. Lysates were diluted to a final cell concentration of approximately 1,000 cells/μL in lysis buffer and transferred to a white opaque flat bottom 384-well-assay plate at approximately 3,000 cells/reaction. Intracellular PKA and CaMKIIα levels were assayed using GloMax 384 Microplate Luminometer with dual injectors (Promega).

### Immunofluorescent Cytochemical Analyses

Cultured human astrocytes were fixed with 1:1 acetone: methanol (V/V) solution following 24 h of treatment with METH, IL-1β or HIV-1. Astrocytes were fixed for 20 min at −20°C and blocked with blocking buffer (2% BSA in 1X PBS containing 0.1% Triton X-100) for 1 h. Cells were then incubated with primary antibodies specific to TAAR1 (1:700, rabbit pAb, Abcam, Cambridge, MA, Cat# ab65633, RRID:AB_1143252, lot GR30601-3), CREB (1:500, rabbit mAb, Cell Signaling Technology Cat# 9197, RRID:AB_331277, lot #16), pCREB^Ser133^ (1:500, rabbit mAb, Cell Signaling Technology Cat# 9198, RRID:AB_2561044, lot #10), pCREB^Ser142^ (1:200, rabbit pAb, Signalway Antibody, College Park, MD, Cat# 11300-2, RRID:AB_1263514, lot #3520) and GFAP (1:400 chicken pAb, Covance, Princeton, NJ, Cat# PCK-591P-100, RRID:AB_291542, lot # D15KF02159) in blocking buffer overnight at 4°C, washed and incubated with Alexa Fluor® secondary antibodies (1:100), anti-rabbit (488 nm, green, Thermo Fisher Scientific Cat# A-11034, RRID:AB_2576217) and anti-chicken (594 nm, red, Thermo Fisher Scientific Cat# A-11042, RRID:AB_2534099). Nuclei were visualized with DAPI (1:800, Cat# D3571, Thermo Fisher Scientific). Micrographs were obtained on an ECLIPSE Ti-4 using the NLS-Elements BR. 3.0 software at room temperature.

### Western Blot Analyses

Non-transfected or transfected astrocytes were cultured as adherent monolayers in 75 cm^2^ flasks at a density of 8 × 10^6^ cells/flask and allowed to recover for 24 or 48 h. Following recovery, cells were treated for 24 h with varying stimuli, and whole cell extracts were isolated using mammalian protein extraction reagent (Cat# 78501, Thermo Fisher Scientific). Cells were collected by scraping in sterile ice-cold PBS to avoid alteration of protein expression on surface of cell membranes. Protein extracts (40 μg) were boiled with 4X NuPAGE lithium dodecyl sulfate loading sample buffer (Cat# NP0007, Thermo Fisher Scientific) at 100°C for 5–10 min, resolved by Bolt 4–12% Bis-Tris gel and subsequently transferred to nitrocellulose membranes using iBlot (Thermo Fisher Scientific). The membranes were incubated with antibodies against PKA [1:700, rabbit pAb, (Cell Signaling Technology Cat# 4782, RRID:AB_2170170, lot #3], pPKA (1:700, rabbit mAb, Cell Signaling Technology Cat# 5661, RRID:AB_10707163, lot # 3), CREB (1:700, rabbit mAb, Cell Signaling Technology Cat# 9197, RRID:AB_331277, lot #16), pCREB^Ser133^ (1:700, rabbit mAb, Cell Signaling Technology Cat# 9198, RRID:AB_2561044, lot #10), CaMKII (1:500, rabbit pAb, Santa Cruz Biotechnology Cat# sc-9035, RRID:AB_634551, lot #E1313), pCaMKII (1:500, mouse mAb, Santa Cruz Biotechnology Cat# sc-32289, RRID:AB_626786, lot #J2913), or pCREB^Ser142^ (1:200, rabbit, Signalway Cat# 11300-2, RRID:AB_1263514, lot #3520) overnight at 4°C, washed and then incubated with anti-rabbit goat antibody IgG conjugated to horseradish peroxidase (1:10,000, Bio-Rad, Hercules, CA, Cat# 170-5046, RRID:AB_11125757) or anti-mouse goat antibody IgG conjugated to horseradish peroxidase (1:10,000, Bio-Rad Cat# 170-5047, RRID:AB_11125753) for 2 h at room temperature. The membranes were then developed with SuperSignal West Femto substrate (Cat# 34095, Thermo Fisher Scientific) and imaged in a Fluorochem HD2 Imager (Protein Simple, Santa Clara, CA). GAPDH (mouse, 1:1000, Santa Cruz Biotechnology Cat# sc-32233, RRID:AB_627679) was used as a loading control.

### Transfection of Astrocytes

Cultured human astrocytes were transfected with On-Target plus® small interfering RNA (siRNA, Dharmacon, Lafayette, CO) pools specific to PKAαβ (siPKAα Cat# L-004649 & siPKAβ, Cat# L−004650), CaMKII (siCaMKII, Cat# L-004942), non-targeting control siRNA pools (siCON, Cat# D-001810), and without siRNA (MOCK) or with pGP-CMV-GCaMP6s, deposited by Douglas Kim (RRID:Addgene_40753) using the Amaxa™ P3 primary cell 96-well-Nucleofector kit and shuttle attachment (Lonza, Walkersville, MD) according to the manufacturer's instructions. Briefly, 1.6 × 10^6^ astrocytes were suspended in 20 μl nucleofector solution containing siCON, siPKAαβ, siCaMKII, (100 nM) or GCaMP6s (0.5 μg/1.6 × 10^6^ cells) and transfected using shuttle protocol CL133. GCaMP6s transfection efficiency averaged approximately 80%. Multiple chambers from the same biological donors were assayed in a minimum of triplicate determinations. Areas were randomly chosen for confocal imaging from each chamber. Baseline green fluorescence suggested successful transfection. Background fluorescence was subtracted from the standardization well for each individual biological donor, to prevent saturation of fluorescence. Transfected cells were supplemented with astrocyte media and incubated for 30 min at 37°C prior to plating. Cells were allowed to recover for 48 h prior to experimental use.

### Confocal Analysis

MOCK- and GCaMP6s-transfected astrocytes were cultured on tissue culture treated μ-slides with a channel height of 0.4 mm (Cat# 86060, Ibidi, Madison, WI) at a density of 1 × 10^5^ cells/chamber in astrocyte media. Prior to live cell imaging, astrocytes were briefly washed with PBS and supplemented with HBSS at 37°C. Time lapse images were obtained every 500 ms, from astrocytes treated with METH (500 μM) or ionomycin (10 μM). Micrographs were obtained on a Carl Zeiss LSM (Jena, Germany). Objective used was 20× Plan-Apochromat, 0.8NA, 0.55 mmWD. PMT photo detection was used with an excitation of 450–490 nm and emission of 593–668 nm. Histogram analysis were performed using ImageJ software; Version: 2.0.0-rc-41/1.5d (Fiji ImageJ Software, the National Institutes of Health, Bethesda, MD) (55). Histogram was generated from fluorescence units obtained at selected time points.

### Ratiometric Calcium Imaging

Primary human astrocytes were seeded at approximately 0.1 × 10^6^ cells on poly-D-lysine coated 22 × 22 × 1 mm coverslips, placed in 6 well-tissue culture dishes and allowed to reach confluency for 24 h. Protocol was modified as previously described (56, 57). Astrocytes were preincubated for 1 h in Krebs–Ringer buffer solution (155 mM NaCl, 2.5 mM CaCl_2_, 1.2 mM MgCl_2_, 24 mM NaHCO_3_, 5 mM KCl, 25 mM HEPES and 5 mM glucose, pH 7.4) containing 3 mM Fura-2-AM (Prokine, Cat# PK-CA707-50033, Heidelberg, Germany) at 37°C prior to METH treatment (500 μM). Coverslips were mounted on laminar-flow perfusion chambers (Warner Instrument, Hamden, CT) mounted on an inverted microscope (Olympus IX81, Olympus, Melville, NY) and attached to a gravity-driven flow-controlled perfusion system (Warner Instrument). Cells were perfused continuously with Krebs-Ringer buffer +/– METH. [Ca^+2^]_i_ was calculated using a Fura-2 calcium calibration standard curve (Thermo Fisher Scientific, Cat# F6774). Basal [Ca^+2^]_i_ measurements were taken following stabilization period of 5 min prior to METH administration, and peak [Ca^+2^]_i_ were measured following METH treatment. Ratiometric data were collected from cells that were alternately illuminated with 340- and 380-nm wavelengths using xenon light source (Lumen200PRO, Prior Scientific, Rockland, MD). The emitted light was captured at 520 nm wavelength using a CCD camera (Hamamatsu camera controller C10600, Hamamatsu Photonics KK, Hamamatsu, Japan). Pixel data were binned (2x2), and images were captured every 3 s. Data were collected and analyzed using commercially available software (Slidebook 5.0, Intelligent Imaging Innovations, Denver, CO).

### Statistical Analyses

Statistical analyses were performed using GraphPad Prism (Version 8.4.0, RRID:SCR_002798) with one-way analysis of variance (ANOVA) and Tukey's *post-test* for multiple comparisons. Linear regression and correlation analysis were performed using Prism with a two-tailed, Pearson correlation coefficient set at 95% confidence interval. *P* ≤ 0.05 were considered statistically significant, and data represent means ± standard error of the mean (SEM).

## Results

### HIV-1 and IL-1β Differentially Regulate Astrocyte TAAR1 and EAAT-2 Levels and Activity

**Figure 1:**
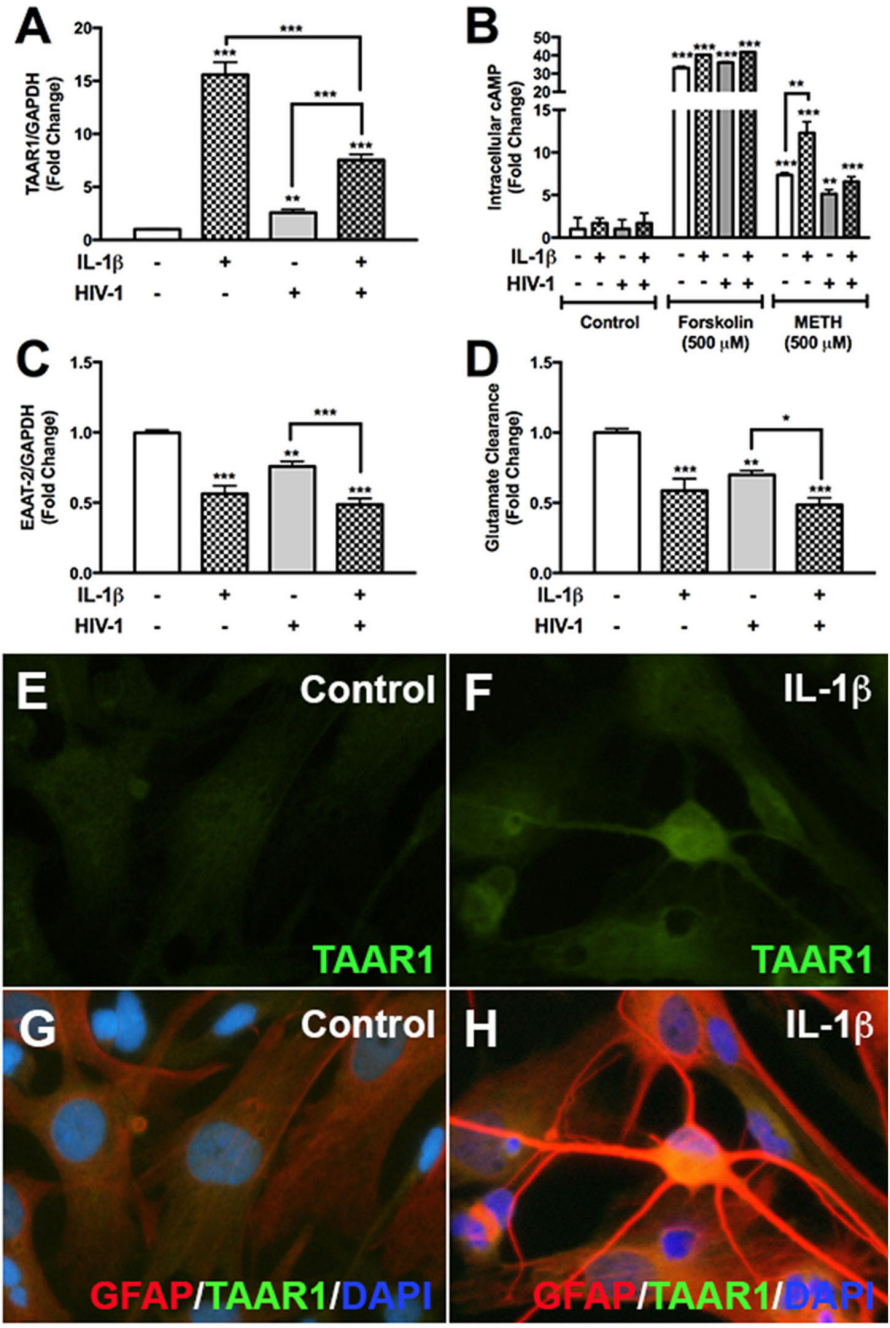
HIV-1 and IL-1β regulate astrocyte TAAR1 & EAAT-2 mRNA levels and function. Primary human astrocytes were treated with HIV-1 (p24 10 ng/mL, gray bars) and IL-1β (20 ng/mL, hatched bars) alone or in combination, and untreated astrocytes were maintained in parallel. TAAR1 mRNA levels were analyzed following treatment with IL-1β ± HIV-1 **(A)**. Intracellular cAMP was quantified to evaluate TAAR1 signaling subsequent to IL-1β ± HIV-1 pretreatment and following 15 min of forskolin or METH stimulation and represented as fold change to control **(B)**. EAAT-2 mRNA levels were evaluated following IL-1β ± HIV-1 treatment **(C)**. Glutamate clearance was measured at 10 h post-glutamate addition **(D)**. Control and IL-1β pretreated cells were fixed and immunostained for glial fibrillary acidic protein (GFAP, red), TAAR1 (green) and DAPI (blue) **(E–H)**. Statistical analyses were performed using GraphPad Prism V6.0 with One-way ANOVA and Tukey's *post-test* for multiple comparisons. *P* ≤ 0.05 were considered statistically significant and data represent means ± SEM. Representative donors were chosen from a minimum of three astrocyte donors each tested and analyzed in a minimum of triplicate determinations (*p < 0.05, **p < *0.01*, ***p < *0.001*).

We have previously demonstrated astrocyte TAAR1 overexpression resulted in a significant decrease in astrocyte EAAT-2 and glutamate clearance (7). Astrocyte TAAR1 knockdown prevented METH-induced EAAT-2 downregulation and increased glutamate clearance (7). Since METH abuse during CNS inflammation and HIV-1 poses greater threat due to their potential to increase TAAR1 levels/activity and crosstalk, we investigated whether IL-1β and HIV-1 downregulated EAAT-2 and affected TAAR1 levels and function (Figure 1). TAAR1 mRNA levels increased significantly with IL-1β (15-fold, ^***^p < *0.001*) and HIV-1 (2.5-fold, ^**^p < *0.01*) alone or in combined treatments (7.5-fold ^***^p < *0.001*; Figure 1A). TAAR1 levels were significantly lower in combined treatments compared to IL-1β but significantly higher compared to HIV-1 (Figure 1A, ^*^^**^p < *0.001*). After IL-1β +/– HIV-1 pretreatment, baseline cAMP levels were unchanged (Figure 1B). Forskolin, a commonly used tool to increase intracellular cAMP levels, significantly increased astrocyte cAMP by ~35-fold, regardless of pretreatments with IL-1β +/– HIV-1 (Figure 1B, ^***^p < *0.001*). METH significantly increased intracellular cAMP in all IL-1β, HIV-1, and IL-1β + HIV-1 pretreated astrocytes (Figure 1B, ^***^p < *0.001*, ^**^p < *0.01*, ^***^p < *0.001*, respectively). However, only IL-1β pretreatment resulted in a significantly increased cAMP response to METH, compared to METH-mediated increases in astrocytes without pretreatment (Figure 1B, ^**^p < *0.01*). As IL-1β and/or HIV-1 increased TAAR1 levels, they also significantly decreased astrocyte EAAT-2 levels in parallel (Figure 1C). IL-1β and HIV-1, alone or in combination, significantly decreased EAAT-2 mRNA levels by 50% (^***^p < *0.001)*, 25% (^**^p < *0.01)*, and 55% (^***^p < *0.001)*, respectively (Figure 1C). Comparisons between treatments showed that IL-1β + HIV-1 was significantly lower compared to HIV-1 alone (Figure 1C, ^***^p < *0.001*). Glutamate clearance, taken as a ratio to MTT activity and converted to a fold change from control, mirrored EAAT-2 mRNA levels (Figure 1D). TAAR1 protein levels increased with IL-1β pre-treatment in astrocytes fixed and immunostained for TAAR1 (green), GFAP (red) and DAPI (blue) (Figures 1E–H). IL-1β mediated a reactive phenotype and increased TAAR1 levels when compared to control astrocytes (Figures 1F,H). Merged images represent TAAR1, GFAP and DAPI overlay (Figures 1G,H). Thus, these data demonstrated that astrocyte TAAR1 expression and intracellular cAMP are elevated in the presence of proinflammatory cytokine, IL-1β, and negatively correlated to astrocyte EAAT-2 (^***^p < *0.001, R*^2^ = 0.92, data not shown).

### METH Activates PKA, Phosphorylating CREB at Serine 133

**Figure 2:**
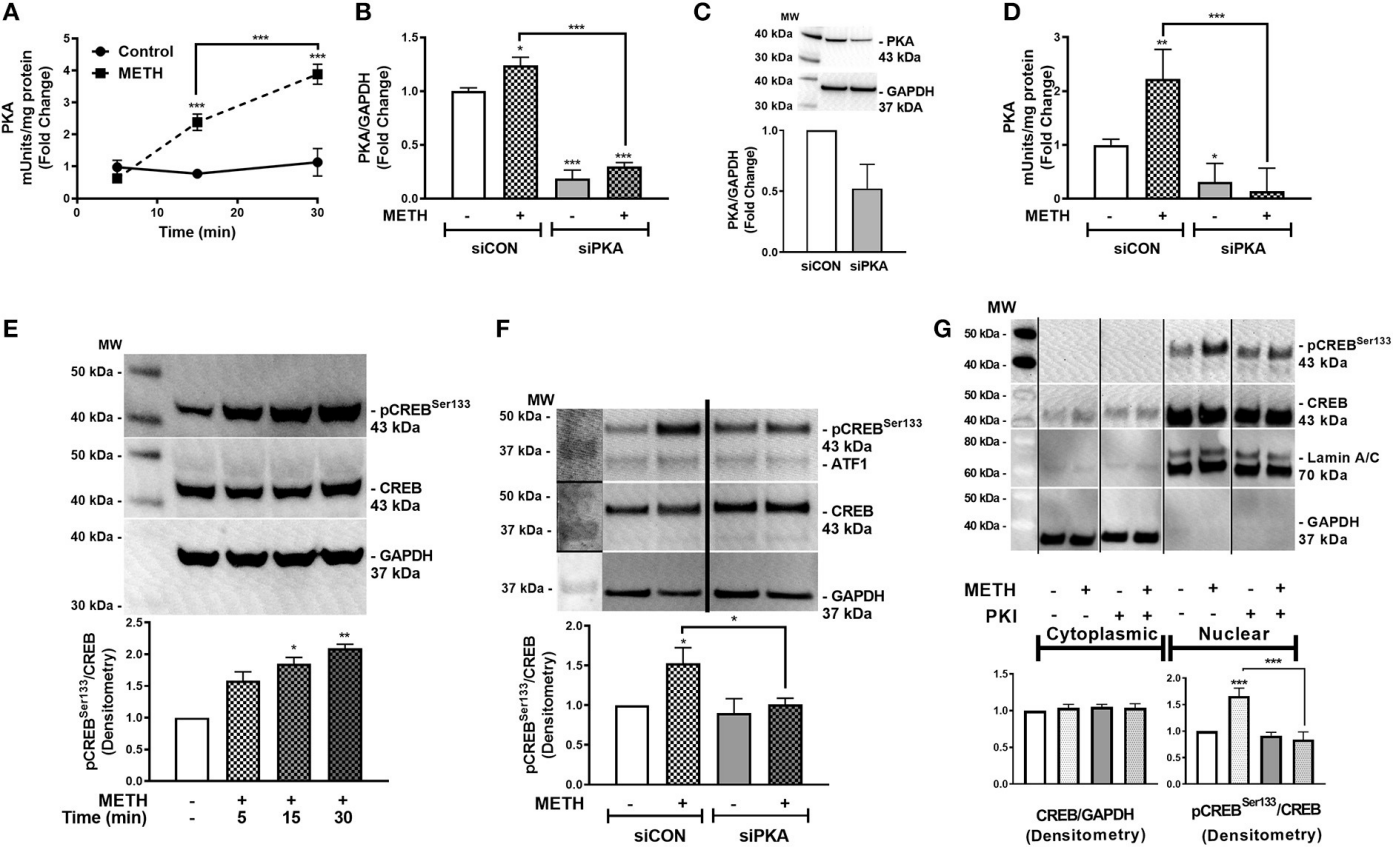
METH activates PKA and phosphorylates CREB at serine 133 (pCREB^Ser133^). PKA enzyme activity in equivalent total cell lysates was quantified at select times post-METH treatment (500 μM, square and hatched lines) and represented as fold changes of PKA mUnits/mg total protein **(A)**. PKA levels and activity were quantified in siCON- and siPKA-transfected astrocytes (clear and gray bars, respectively, as shown by fold changes in PKA/GAPDH mRNA **(B)**, protein **(C)** and PKA activity (mUnits/mg total protein) **(D)**. Immunoblotting for METH induction of pCREB^Ser133^ to total CREB over time was assayed by western blot with detected bands at 43 kDa **(E)**. To determine METH-induced pCREB^Ser133^ via PKA, total cell lysates were collected at 30 min post-METH treatment in siCON- and siPKA-transfected astrocytes and immunoblotted for pCREB^Ser133^ and total CREB. Bands are detected at 43 kDa for pCREB^Ser133^ and total CREB **(F)**. Cytoplasmic and nuclear protein extracts were collected from astrocytes treated with PKI (gray bars) +/– METH (hatched bars) and immunoblotted for pCREB^Ser133^ **(G)**. The same blot is represented in panel F & G from different sections; dividing lines represent cut sections. Representative western blots are shown in **(E–G)**. Densitometry analyses were performed to quantify band intensities of phospho-proteins to total proteins on multiple immunoblots and represented as fold changes to control ± SEM, in respective panels [**(E–G)**, *n* = 3]. **(A–D)** is a representative donor chosen from multiple individual biological astrocyte donors that were tested; each was analyzed in a minimum of triplicate determinations. Molecular weight markers are identified on each western blot (MW) (*p < 0.05, **p < 0.01, ***p < *0.001*).

METH increases intracellular cAMP in primary human astrocytes (7). cAMP, as a secondary messenger, mediates canonical signal transduction pathways that activate PKA and lead to downstream phosphorylation of substrates, including CREB, at serine 133 (58). Therefore, we next investigated PKA activity in primary human astrocytes following METH treatment, represented as fold changes +/– SEM (Figure 2A). PKA activity did not change in control astrocytes over time; however, METH significantly increased PKA activity at 15 min that continued to significantly increase at 30 min (Figure 2A, ^***^p < *0.001*). PKA mRNA levels significantly decreased in astrocytes transfected with siPKA compared to siCON-transfected astrocytes, with/out METH treatment (Figure 2B, ^***^p < *0.001*). In parallel, total PKA levels decreased 50% in siPKA-transfected astrocytes compared to siCON-transfected astrocytes (Figure 2C). METH induced a significant increase in PKA activity in siCON-transfected astrocytes (^**^p < *0.01*) that was significantly reduced with PKA RNAi (Figure 2D, ^***^p < *0.001*). Furthermore, baseline PKA activity was lower in siPKA-transfected astrocytes reflecting decreases in baseline PKA (Figure 2D, ^*^p < *0.05*). Western blots confirmed METH-induced phosphorylation of CREB at serine 133 (pCREB^Ser133^) (Figure 2E). Densitometry analyses, from four independent biological donors, showed METH to significantly induce pCREB^Ser133^ in 15 min (Figure 2E, ^*^p < *0.05*) that remained elevated at 30 min (Figure 2E, ^**^p < *0.01*). METH-mediated phosphorylation of CREB at serine 133 increased in siCON-transfected astrocytes at 30 min (Figure 2F, ^*^p < *0.05)* that was significantly lower in siPKA-transfected astrocytes (Figure 2F, ^*^p < *0.05*). Constitutively phosphorylated CREB at serine 133 is localized in the nucleus, and nuclear pCREB^Ser133^ is significantly increased following METH treatment at 30 min (Figure 2G, ^***^p < *0.001*). METH- mediated pCREB^Ser133^ was prevented by cAMP-dependent PKA inhibitor, PKI, by ~60% (Figure 2G, ^***^p < *0.001*) implying pCREB^Ser133^ is *via* METH-induced PKA activation.

### METH Transiently Increases Intracellular Calcium in GCaMP6s-Transfected Astrocytes

**Figure 3:**
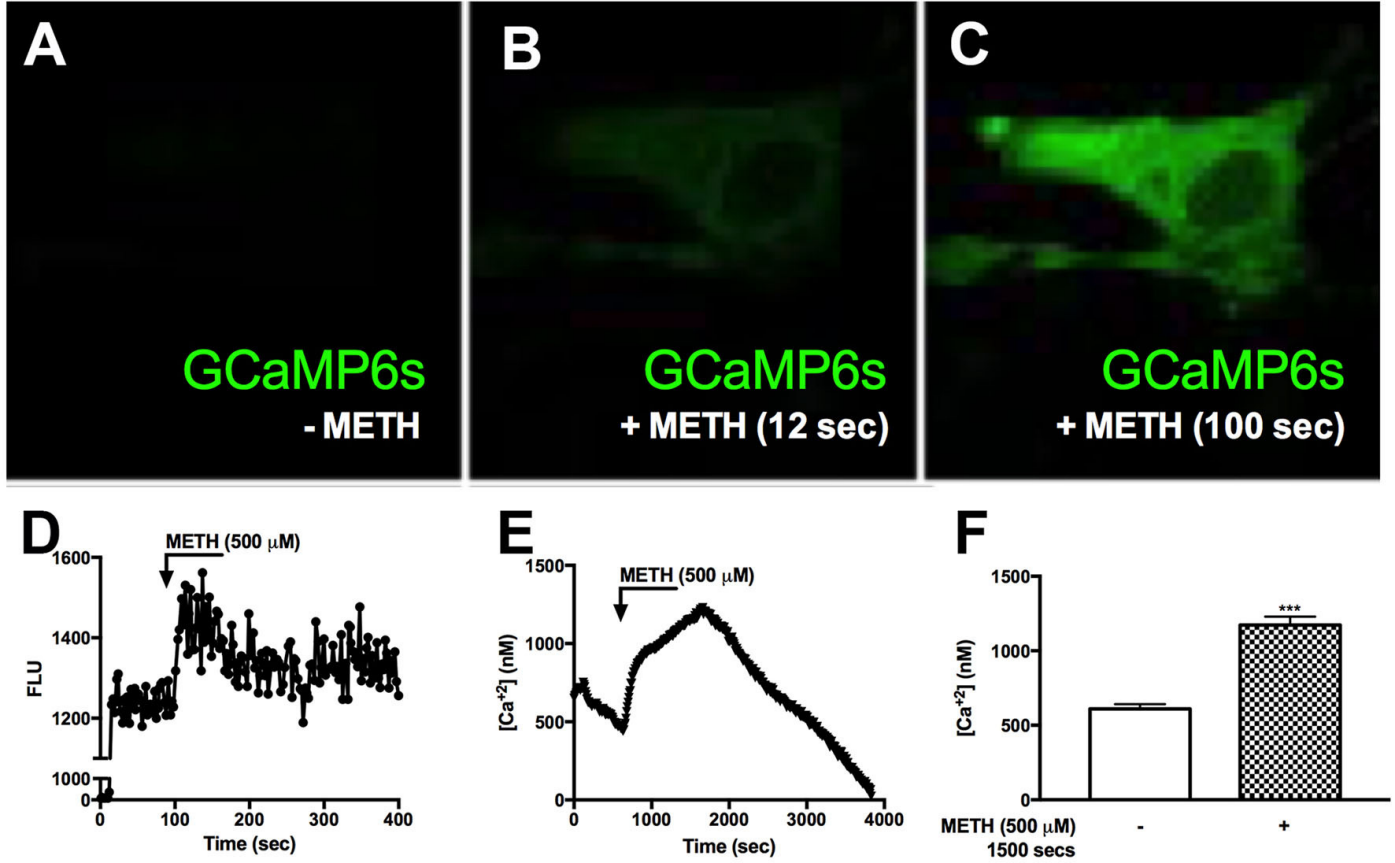
METH induces intracellular calcium signaling in GCaMP6s-transfected astrocytes. Primary human astrocytes were transfected with GCaMP6s, a plasmid expressing an ultrasensitive protein calcium sensor, and allowed to recover overnight. MOCK-transfected astrocytes were maintained as controls in parallel. Transfected cells were treated with METH (500 μM, hatched bars) and ionomycin (10 μM) as a positive control [images not shown, **(A–C)**]. Fluorescence was visualized by confocal microscopy, and images were captured every 500 msec. **(A–C)** depict images taken from one specific cell prior to METH addition **(A)**, and post-METH addition at 12 and 100 s, respectively **(B,C)**. The histogram shows cumulative data of the pictured astrocyte captured over the entire imaging period **(D)**. [Ca^+2^]_i_ were quantified with a Fura-2 standard curve, representing changes in emission at 340 and 380 nm at an excitation of 510 nm (image not shown). Absolute [Ca^+2^]_i_ are represented in **(E,F)**. **((A–E))** is a single astrocyte traced over time representing average changes **(E)**. Several astrocytes (*n* = 15) are represented as a bar graph **(F)**. **(A–E)** are a single cell representing average change of multiple astrocyte donors that were tested; each was analyzed in a minimum of triplicate determinations. ***p < *0.001*.

Reports suggest that METH results in increased [Ca^+2^]_i_ in neurons (27, 59). To determine METH-induced activation of [Ca^+2^]_i_ stores, primary human astrocytes were transfected with a GCaMP6s plasmid (60). Baseline fluorescence was measured in the absence of external stimuli and plotted as 0 FLU (Figures 3A,D), demonstrating undetectable [Ca^+2^]_i_, i.e., no fluorescence (Figure 3A). There was an approximate 80% transfection efficiency for GCaMP6s transfection in primary human astrocytes. Although low fluorescence was detectable following 12 s of METH treatment (Figure 3B), a robust increase in fluorescence was visualized at 100 s (Figure 3C). FLU represents fluorescence from imaged astrocyte over time (Figure 3D). These data support the innovative use of GCaMP6s-transfected astrocytes in visualizing METH-induced increases of intracellular calcium via live cell imaging. To further support METH-mediated increases of [Ca^+2^]_i_, astrocytes were treated with Fura-2-AM and stimulated with METH, and subsequent calcium levels were quantified (Figures 3E,F). Baseline measurements were taken for 5 min prior to METH stimulation. METH (500 μM) raised intracellular calcium concentrations by ~750 nM, reaching 1,000–1.250 nM [Ca^+2^]_i_ (Figure 3E, representative donor). Averages from 15 individual astrocytes show basal [Ca^+2^]_i_ to be ~500 nM with significant increases of [Ca^+2^]_i_ mediated by METH (Figure 3F, ^***^p < *0.001*). These data confirm that increases of [Ca^+2^]_i_ are sufficient to activate downstream signaling cascades and phosphorylation of substrates.

### METH Activates CaMKII, Phosphorylating CREB at Serine 142

**Figure 4:**
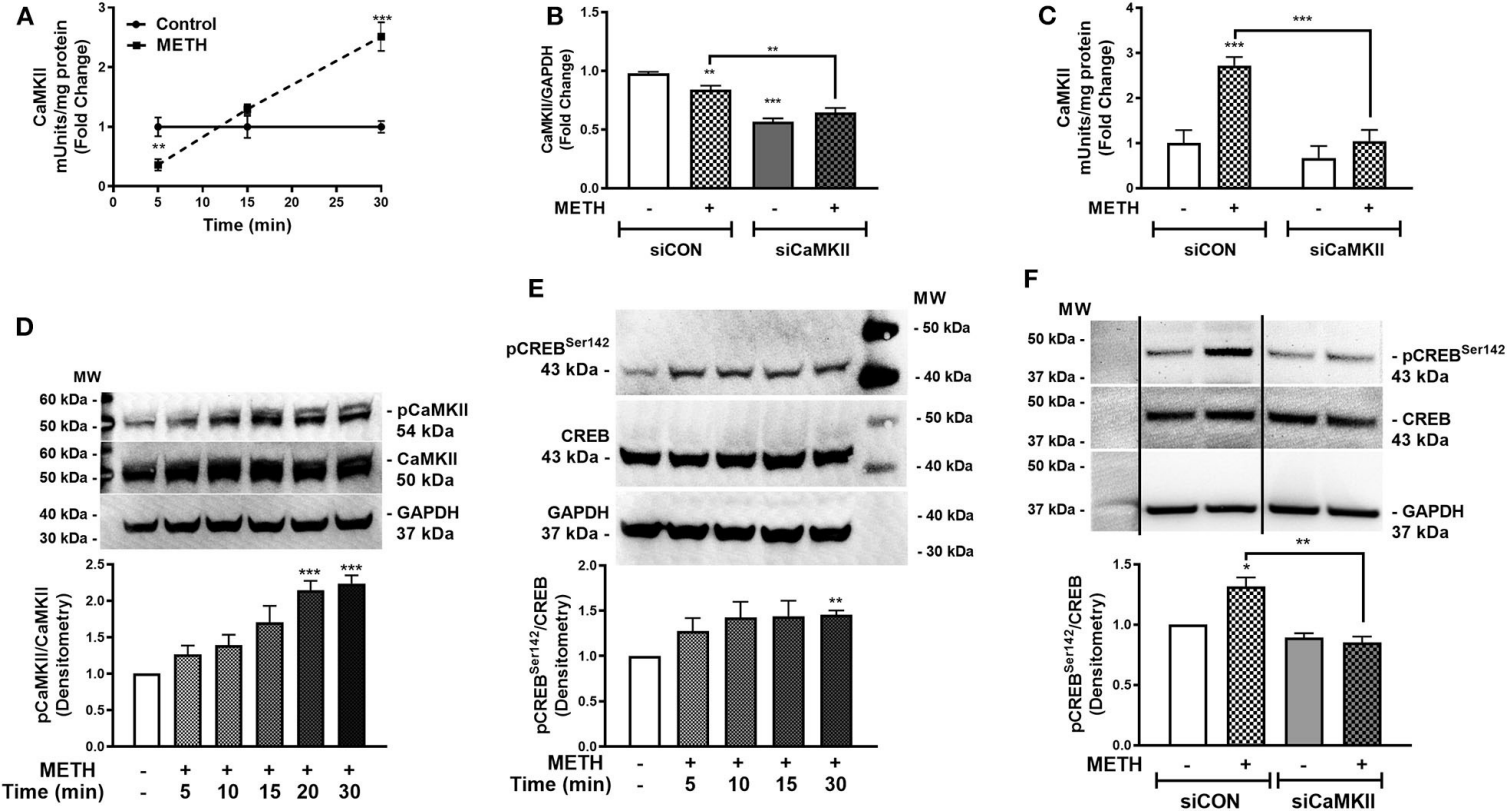
METH activates CaMKII and phosphorylates CREB at serine 142 (pCREB^Ser142^). CaMKII enzyme activity was quantified at 5, 15, and 30 min post-METH treatment (500 μM, squares and hatched bars) in equal amounts of cell lysates and represented as fold changes of CaMKII mUnits/mg total protein **(A)**. CaMKII levels and activity were quantified in siCON- and siCaMKII-transfected astrocytes (clear and gray bars, respectively), and represented as CaMKII/GAPDH fold changes and fold changes of CaMKII mUnits/mg total protein **(B,C)**. To evaluate METH-induced phosphorylation of CaMKII, immunoblotting for pCaMKII total CaMKII and GAPDH was detected at 54, 50, and 37 kDa, respectively in astrocytes treated with METH (500 μM) at different time points **(D)**. Immunoblotting for pCREB^Ser142^ to total CREB over time was assayed by western blot with detected bands at 43 kDa **(E)**. To determine METH-induced pCREB^Ser142^ via CaMKII, total cell lysates were collected at 30 min post-METH treatment in siCON- and siCaMKII-transfected astrocytes and immunoblotted for pCREB^Ser142^ and total CREB **(F)**. The same blot is represented in **(F)** from different sections, dividing line represents a cut section. Representative donors are shown in all panels from multiple astrocyte donors that were tested; each was analyzed in a minimum of triplicate determinations. Densitometry analyses of ratio for phospho-proteins to total proteins was performed to quantify band intensities on multiple immunoblots and represented as fold changes to control ± SEM [**(D–F)**, *n* = 3]. Molecular weight markers are identified on each western blot (MW) (*p < 0.05, **p < *0.01*, ***p < *0.001*).

Activation of CaMKII is sensitive to increases in [Ca^+2^]_i_ (47). We showed METH increases [Ca^+2^]_i_ in primary human astrocytes (Figure 3). Thus, we investigated CaMKII activity in primary human astrocytes following METH treatment (Figure 4A). CaMKII activity did not change in control astrocytes over time. METH significantly reduced CaMKII activity as early as 5 min (Figure 4A, ^**^p < *0.01*) that increased at 15 min and was significantly higher than control at 30 min (Figure 4A, ^***^p < *0.001*). CaMKII mRNA levels significantly decreased in siCaMKII-transfected astrocytes by approximately 50% +/- METH treatment (Figure 4B, ^***^p < *0.001*, ^**^p < *0.01*). METH treatment alone significantly reduced CaMKII levels in siCON-transfected astrocytes (Figure 4B, ^**^p < *0.01*). METH significantly increased CaMKII activity in siCON- transfected astrocytes (Figure 4C, ^***^p < *0.001*). However, CaMKII activity was ~2-fold lower in siCaMKII-transfected astrocytes as compared to siCON-transfected astrocytes (Figure 4C, ^***^p < *0.001*). Immunoblotting confirmed METH-induced phosphorylation of CaMKII over time, detected as pCaMKII at 54 kDa (Figure 4D). Densitometry analyses from three independent biological donors confirmed METH induced significant increases in pCaMKII at 20 and 30 min (Figure 4D, ^***^p < *0.001*). METH treatment over time mediated pCREB^Ser142^ that was significantly increased at 30 min (Figure 4E, ^**^p < *0.01*). METH significantly increased pCREB^Ser142^ in siCON-transfected astrocytes (Figure 4F, ^*^p < *0.05*). CaMKII RNA interference abrogated METH-induced pCREB^Ser142^ compared to siCON (Figure 4F, ^**^p < *0.01*) implying pCREB^Ser142^ is via METH-induced CaMKII activation. Taken together, METH mediates CREB phosphorylation at serine 142 via CaMKII activation.

### TAAR1 Selective Antagonist, EPPTB, Blocks METH-Induced Signaling in Astrocytes

**Figure 5:**
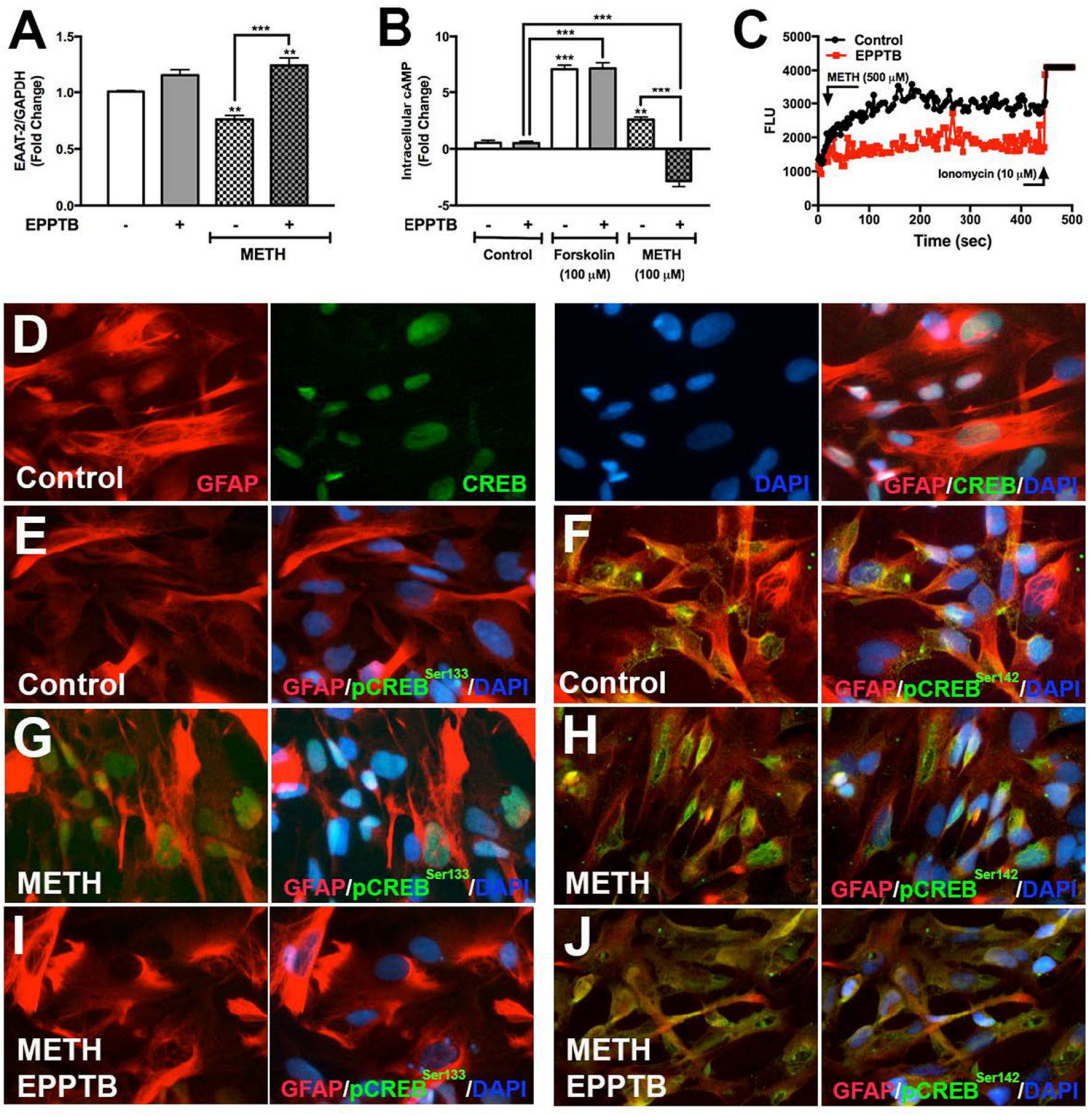
TAAR1 selective antagonist, EPPTB, blocks METH-induced signaling in astrocytes. EAAT-2 mRNA levels were assayed in RNA isolated at 8 h post METH (hatched bars) +/– EPPTB (20 μM, gray bars), a TAAR1 selective antagonist **(A)**. Changes in cAMP levels were quantified in astrocytes treated with EPPTB for 1 h prior to METH treatment (100 μM) or forskolin treatment (100 μM) and represented as fold changes ± SEM **(B)**. GCaMP6s-transfected astrocytes were pretreated with EPPTB (red) for 1 h prior to METH stimulation in parallel to untreated astrocytes. Increases in [Ca^+2^]_i_ were visualized by confocal microscopy and represented as a histogram over time **(C)**. Astrocytes treated with METH, +/– EPPTB pretreatment, were fixed and immunostained with antibodies specific for total CREB [green, **(D)**], pCREB^Ser133^ [green, **(E,G,I)**] or pCREB^Ser142^ [green, **(F,H,J)**], GFAP (red) and DAPI [blue, **(D–J)**]. Statistical analyses were performed using GraphPad Prism V6.0 with One-way ANOVA and Tukey's *post-test* for multiple comparisons. *P* ≤ 0.05 were considered statistically significant, and data represent ± SEM. Representative donors chosen from multiple astrocyte donors were tested; each was analyzed in a minimum of triplicate determinations (**p < *0.01*, ***p < *0.001*).

EPPTB is a selective high affinity antagonist for TAAR1 in neurons, potently antagonizing TAAR1-induced cAMP accumulation in HEK293 cells (40). There are no studies describing EPPTB antagonism of astrocyte TAAR1 activity and/or regulation of astrocyte EAAT-2. Therefore, we evaluated EPPTB effects on astrocyte EAAT-2 (Figure 5A). There were no changes in astrocyte EAAT-2 with EPPTB alone. As previously observed, METH significantly reduced astrocyte EAAT-2 mRNA that significantly increased with EPPTB pretreatment (Figure 5A, ^**^p < *0.01*, ^***^p < *0.001*, respectively). The effects of EPPTB on METH-mediated TAAR1 activation were quantified by measuring intracellular cAMP (Figure 5B). EPPTB pretreatment did not significantly affect forskolin-induced intracellular cAMP (^***^p < *0.001*). On the other hand, increased cAMP, mediated by METH, was significantly reduced with EPPTB pretreatment (Figure 5B, ^***^p < *0.001*). Additionally, TAAR1 antagonism with EPPTB prevented METH-mediated increases of [Ca^+2^]_i_ (Figure 5C). Together, these data suggest that successful antagonism of TAAR1 with EPPTB prevented METH-induced TAAR1 activation and prevented METH-mediated EAAT-2 downregulation. To investigate METH-induced CREB phosphorylation *via* TAAR1, astrocytes pretreated with EPPTB, and activated with METH (500 μM), were fixed and immunostained for CREB (green), pCREB^Ser133^ (green), pCREB^Ser142^ (green), GFAP (red), and DAPI (blue) (Figures 5D–J). In control astrocytes, total CREB was primarily within the nucleus (Figure 5D). CREB phosphorylated at serine 133 in control astrocytes was low (Figure 5E). CREB phosphorylated at serine 142 showed to be primarily localized perinuclear in the absence of stimulation (Figure 5F). Upon METH treatment, pCREB^Ser133/142^ appeared more robust in astrocyte nucleus, as seen localized with DAPI (Figures 5G,H). EPPTB pretreatment blocked METH-induced nuclear localization of both pCREB^Ser133^ and pCREB^Ser142^ (Figures 5I,J). Thus, these data demonstrate that EPPTB successfully antagonizes astrocyte TAAR1 preventing METH-induced activation and CREB phosphorylation at serine 133 and 142, ultimately preventing EAAT-2 regulation.

### IL-1β and HIV-1 Activate CREB

**Figure 6:**
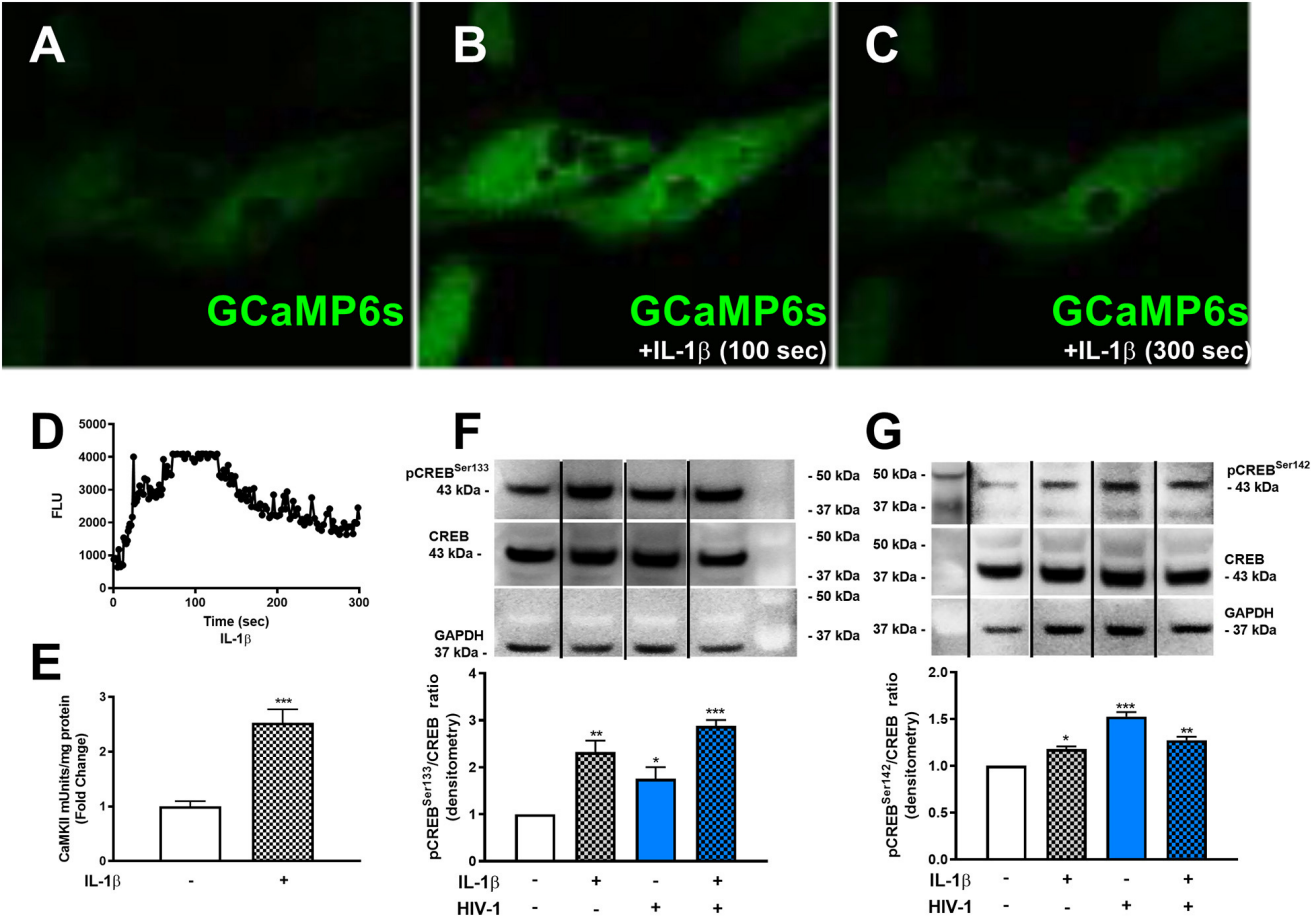
HAND-relevant stimuli increase [Ca^+2^]_i_, CaMKII activity and phosphorylation of CREB at serine 133 & 142. Primary human astrocytes were transfected with GCaMP6s. MOCK-transfected astrocytes were maintained as controls in parallel. Transfected cells were treated with IL-1β (20 ng/mL, **A–C**) or ionomycin (10 μM) as a positive control (images not shown). Fluorescence was visualized by confocal microscopy, and images were captured every 500 ms for 5 min. **(A–C)** depict astrocytes treated with IL-1 at selected time points. The histogram shows cumulative data, captured over 5 min **(D)**. CaMKII activity was measured in astrocytes stimulated with IL-1β (20 ng/mL, hatched bars) for 30 min **(E)**. Primary human astrocytes were treated with IL-1β and HIV-1 (blue bars), alone and in combination. Protein lysates were collected 30 min post-treatment and immunoblotted for total CREB, pCREB^Ser133^, pCREB^Ser142^, and GAPDH **(F,G)**. Dividing lines on blots represented in **(F,G)** represent cut sections from the same blot. Representative western blots are shown in **(F,G)**. Densitometry analyses were performed to quantify band intensities on multiple immunoblots and represented as fold changes to control ± SEM, in [**(F,G**), *n* = 3]. Statistical analyses were performed using GraphPad Prism V6.0 with one-way ANOVA and Tukey's *post-test* for multiple comparisons. *P* ≤ 0.05 were considered statistically significant, and data represent means ± SEM. This figure depicts representative donors chosen from multiple astrocyte donors that were tested; each was analyzed in a minimum of triplicate determinations. Data are shown as cumulative fold changes. *n* represents individual biological replicates. Molecular weight markers are identified on each western blot (MW) (*p < *0.05*, **p < *0.01*, ***p < *0.001*).

Although METH phosphorylated the co-activating form of CREB, astrocyte EAAT-2 remains downregulated. Likewise, IL-1β and HIV-1 resulted in EAAT-2 downregulation regardless of four NF-κB elements in the EAAT-2 promoter. To address this conundrum and determine if IL-1β and HIV-1 signal similarly to activate the dominant repressor form of CREB, pCREB^Ser142^, we measured [Ca^+2^]_i_ changes following IL-1β treatments. IL-1β led to increased [Ca^+2^]_i_ in GCaMP6s-transfected astrocytes (Figures 6A–D). Fluorescence increased as early as 100 s of IL-1β treatment and began quenching at 150 s (Figures 6B,D). The change in FLU is plotted as a histogram in (Figure 6D). Furthermore, IL-1β significantly increased CaMKII activity at 30 min by 2.5-fold (Figure 6E, ^***^p < *0.001*). Western blots showed IL-1β and HIV-1 induced CREB phosphorylation at serine 133 and 142 (Figures 6F,G). Densitometry analyses, from three independent donors, confirmed IL-1β and HIV-1 alone, and in combination, significantly induced pCREB^Ser133^ at 30 min (Figure 6F, ^**^p < *0.01*, ^*^p < *0.05*, ^***^p < *0.001*, respectively). Likewise, IL-1β and HIV-1 alone and in combination significantly induced pCREB^Ser142^ at 30 min (Figure 6G, ^*^p < *0.05*, ^***^p < *0.001*, ^**^p < *0.01*, respectively). Taken together, these data demonstrate IL-1β and HIV-1 induced both the co-activating and co-repressor forms of CREB.

### Astrocyte EAAT-2 Is Regulated by METH-Mediated Activation of CaMKII, Not PKA

**Figure 7:**
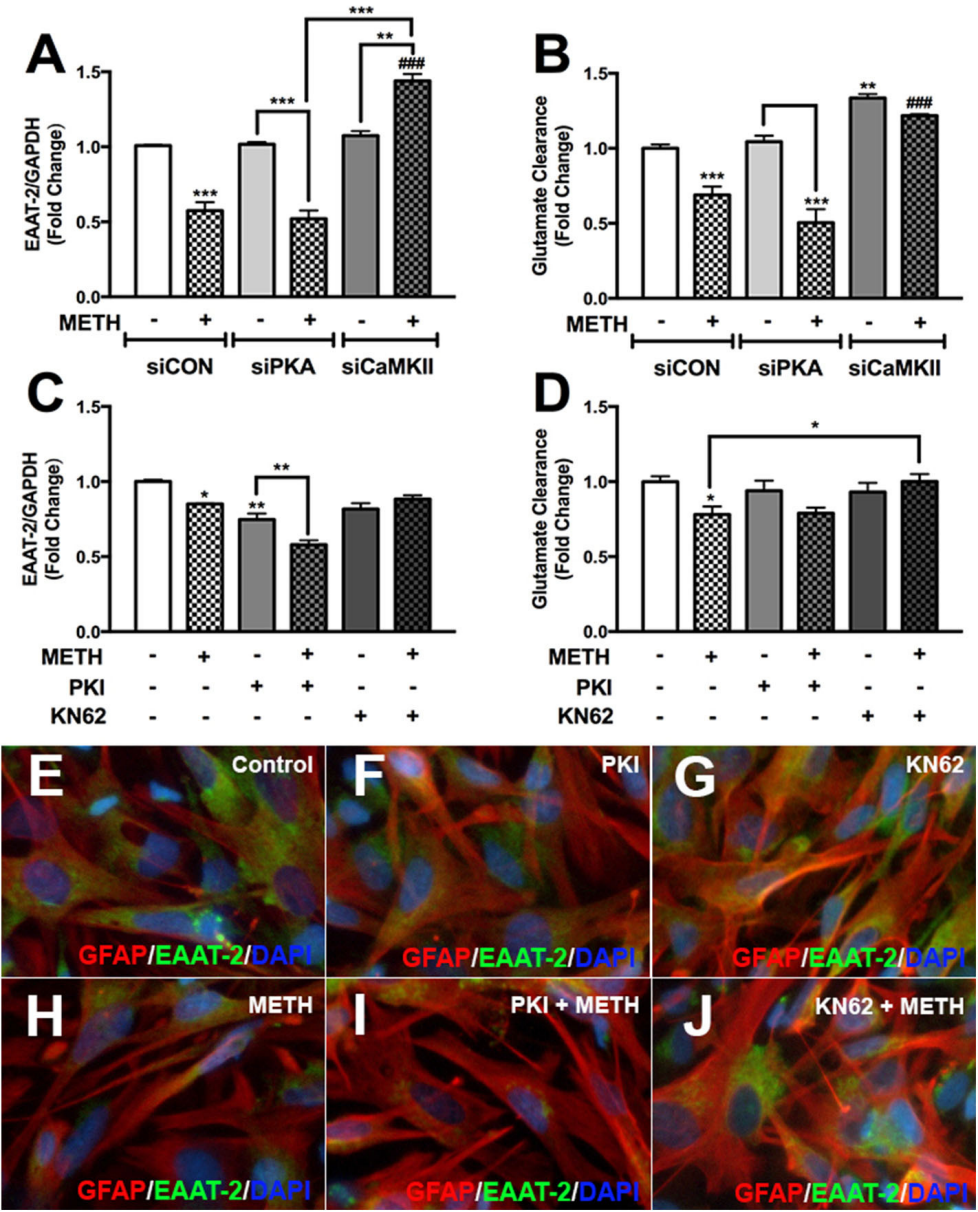
METH-induced activation of PKA and CaMKII differentially regulates astrocyte EAAT-2. Cultured human astrocyte transfected with siCON (clear bars), siPKA (light gray bars), and siCaMKII (dark gray bars) were treated with METH (500 μM, hatched bars). Alternatively, astrocytes were pretreated with cAMP-dependent PKA inhibitor, PKI (light gray bars), or CaMKII inhibitor, KN62 (dark gray bars), for 1 h prior to METH treatment (hatched bars). RNA was collected from transfected and pretreated astrocytes, and EAAT-2 mRNA levels were analyzed 8 h post-METH treatment **(A,C)**. Glutamate clearance was quantified at 10 h post-glutamate addition **(B,D)**. Astrocytes pretreated with PKA and CaMKII inhibitor +/– METH treatment were immunostained for GFAP (red), EAAT-2 (green), or DAPI [blue, **(E–J)**]. Statistical analyses were performed using GraphPad Prism V6.0 with one-way ANOVA and Tukey's *post-test* for multiple comparisons. *P* ≤ 0.05 were considered statistically significant, and data represent means ± SEM. This figure depicts representative donors chosen from multiple astrocyte donors that were tested; each was analyzed in a minimum of triplicate determinations. Data are shown as cumulative fold changes (*p < *0.05*, **p < *0.01*, ***p < *0.001*, ^*###*^*p* < *0.001*).

We have demonstrated METH activates PKA/pCREB^Ser133^ and CaMKII/pCREB^Ser142^ signal transduction pathways in primary human astrocytes. To further evaluate the impact of PKA/CREB^Ser133^ and CaMKII/CREB^Ser142^ on EAAT-2 regulation we transfected astrocytes with siRNAs targeting PKA and CaMKII or pretreated astrocytes with PKA or CaMKII inhibitors prior to METH treatment; as they are the downstream substrate targets for secondary messengers, cAMP and [Ca^+2^]_i_. METH mediated EAAT-2 downregulation and decreased glutamate clearance activity in siCON-and siPKA-transfected astrocytes (Figures 7A,B, ^***^p < *0.001)*. CaMKII downregulation did not change EAAT-2 levels but significantly increased glutamate clearance alone (Figures 7A,B, ^**^p < *0.01*). METH significantly increased EAAT-2 levels in siCaMKII-transfected astrocytes compared to siCON + METH and siPKA + METH (Figure 7A, ^*###*^*p* < *0.001*) or to siCaMKII transfection alone (Figure 7A, ^**^p < *0.01*). Likewise, glutamate clearance was significantly higher following METH treatment in siCaMKII-transfected astrocytes compared to that in siCON- and siPKA-transfected astrocytes (Figure 7B, ^*###*^*p* < *0.001*). PKI significantly reduced EAAT-2 mRNA levels alone or with METH (Figure 7C, ^**^p < *0.01*), but did not change astrocyte glutamate clearance regardless of METH treatment (Figure 7D). CaMKII inhibitor, KN62, prevented METH-mediated EAAT-2 downregulation (Figure 7C) and significantly increased astrocyte glutamate clearance following METH treatment (Figure 7D, ^*^p < *0.05*). Astrocytes pretreated with PKI or KN62, with or without METH, were fixed and immunostained for EAAT-2 (green), GFAP (red), and DAPI (blue) (Figures 7E–J). Astrocyte EAAT-2 protein levels did not robustly change with PKI or KN62 alone compared to control astrocytes (Figures 7E–G). METH reduced EAAT-2 protein expression alone or in combination with PKI (Figures 7H,I). However, pretreatment with KN62 inhibited EAAT-2 downregulation by METH (Figure 7J). Taken together, our data show compelling evidence that [Ca^+2^]_i_/CaMKII/pCREB^Ser142^ is the predominate pathway resulting in METH-induced EAAT-2 downregulation.

## Discussion

This study uncovers critical signaling pathways in astrocytes mediated via METH-induced TAAR1 activation. Importantly, we identify CREB as a master regulator of astrocyte EAAT-2 following METH treatment. We demonstrate METH activates canonical cAMP/PKA/pCREB^Ser133^ and [Ca^+2^]_i_/CaMKII/pCREB^Ser142^ signaling in primary human astrocytes, downstream of TAAR1. TAAR1 is established to trigger an accumulation of intracellular cAMP (24), regulating expression, localization and function of monoamine transporters *via* phosphorylation of PKA and PKC (61–64). Activation of kinases such as PKA, results in phosphorylation of substrates including CREB. CREB phosphorylation traditionally involves transcriptional activation of CREB binding genes (65). Recruitment of CREB to the EAAT-2 promoter suggests increased promoter activity and EAAT-2 upregulation (29, 66). This study identifies that METH phosphorylates CREB at serine 133; however, EAAT-2 transcription decreases, indicating a differential role for CREB in EAAT-2 regulation, downstream of TAAR1. We tested the hypothesis that TAAR1-mediated signaling, following METH stimulation, dually triggers the activating and dominant repressing forms of CREB, thus dictating EAAT-2 downregulation. We demonstrate astrocyte TAAR1 levels increase following IL-1β and HIV-1 treatment, suggesting a broader role for astrocyte TAAR1 during neuroinflammation. Taken together, our studies reveal a delicate balance between METH-induced activation of cAMP/PKA/pCREB^Ser133^ and [Ca^+2^]_i_/CaMKII/pCREB^Ser142^ signaling in the regulation of astrocyte EAAT-2, which tips the downstream balance of EAAT-2 function from glutamate uptake to excitotoxicity.

In this study we confirm that following METH treatments, intracellular cAMP increases, activating PKA, and phosphorylating CREB at serine 133, yet results in astrocyte EAAT-2 downregulation. Kim et al. showed that exogenous application of dibutyl cAMP increases EAAT-2 transcription supporting our hypothesis that selectively activating cAMP/PKA/pCREB^Ser133^ pathway is enough to prevent METH-mediated EAAT-2 downregulation (66). Decreases in astrocyte EAAT-2, subsequent to METH treatment, is not prevented with PKA downregulation, indicating METH responses in astrocytes are likely activating transcriptional repressors. Alternatively, the abundance and distribution of cAMP-regulated guanine nucleotide exchange factors (Epac/cAMP-GEF) warrants further studies, since activation of this exchange protein may converge synergistically with PKA, or mediate increases of [Ca^+2^]_i_ to regulate other biological functions [reviewed in (67)]. Nonetheless, we demonstrate TAAR1 activation increases [Ca^+2^]_i_ in primary human astrocytes in parallel to cAMP. Several lines of evidence suggest CaMKs, downstream of increased [Ca^+2^]_i_, activate CREB, and result in transcriptional activation and/or repression (68). This balance is mediated via phosphorylation of serine 133 and 142 (36, 37). Phosphorylation of CREB at serine 142 acts as a dominant negative regulator of pCREB^Ser133^-induced transcriptional activation, despite significantly higher amounts of pCREB at serine 133 vs. serine 142 (37, 68). Efficient binding of pCREB^Ser133^ to promoter elements continues; however, pCREB^Ser142^ prevents CBP dimerization, thus inhibiting CREB-supported transcription (68). We hypothesize that the absence of the dominant repressor, pCREB^Ser142^, would permit transcriptional activation of EAAT-2. Consistent with increased EAAT-2 transcription following dibutyl cAMP application that selectively activates PKA/pCREB^Ser133^, we propose that forfeiting CaMKII/pCREB^Ser142^-induced transcriptional repression, while activating PKA/pCREB^Ser133^ is a mechanistic strategy for increasing EAAT-2.

Recently Kumar et al. demonstrated that METH exposure decreased pCaMKII levels in several brain regions of HIV tat transgenic mice (69). These changes correlated with decreased working and spatial memory, neurotrophin levels, and decreased synaptodendritic integrity. Autophosphorylation of CaMKII at threonine 286 is required for kinase activity (70). Both HIV and METH have been shown to reduce pCaMKII in rodents and SIV in rhesus macaques (71, 72). These changes have been associated with neuronal responses and have not been evaluated in astrocytes. Here we show that METH significantly reduced CaMKII activity at 5 min and increased activity by 30 min in human astrocytes. Further, METH, HIV-1, and IL-1β increased pCREB^Ser142^ phosphorylation, which was associated with total CaMKII levels and activity. Together, this suggests that CaMKII activity may be regulated differently in neurons and astrocytes by METH and HIV.

Antagonizing astrocyte TAAR1 with EPPTB, blocked METH-mediated increases in cAMP and [Ca^+2^]_i_, prevented METH-induced phosphorylation of CREB at serine 133/142 and averted METH-mediated EAAT-2 decreases. In fact, in EPPTB pretreated astrocytes, METH significantly reduced cAMP levels below baseline, reflecting decreased ATP levels, and potential dysregulation of cellular energy. Inhibiting and/or antagonizing TAAR1, with EPPTB, may be enough to prevent METH-induced EAAT-2 downregulation; yet, METH abuse during CNS inflammation and HIV-1 poses greater threat due to their potential to increase TAAR1 levels/activity and crosstalk between PKA, CaMKII and NF-κB. For instance, the rate of NF-κB translocation into the nucleus is regulated by PKA-induced phosphorylation of NF-κB/Rel complexes, downstream of cAMP (73). Interestingly, NF-κB activation of target genes is optimized by interaction of the RelA subunit with CREB co-activators, CBP and p300 (74, 75). Furthermore, the region of pCREB^Ser133^ that interacts with CBP also interacts with RelA, thereby inhibiting NF-κB activity (76). This serves as compelling evidence for CREB and NF-κB mechanistic crosstalk, ultimately influencing how they regulate transcription of target genes in astrocytes. IL-1β and HIV-1 initiate similar signaling mechanisms in astrocytes as METH, independent of TAAR1 activation. The additive effects of IL-1β and HIV-1 on pCREB^Ser133^ vs. inhibitory effects on pCREB^Ser142^ need to be further investigated and may be a promising mechanistic intervention to prevent glutamate excitotoxicity during neuroinflammatory disorders. Targeting downstream of CaMKII, to prevent pCREB^Ser142^, has larger implications for all genes with CREB promoter elements in astrocytes including inflammatory mediators, oxidative stress genes and growth factors (77–81).

Non-functional mutations in mouse TAAR1 affect METH intake, hypothermia and conditioned taste aversion (82, 83). Humans possess many TAAR1 variants with sensitivity to ligands (84–87). However, the effects of TAAR1 variants on signaling cascades and risk for MUD, HAND, and neuropsychological disease will need to be evaluated. Additionally, there is an apparent dichotomy for TAAR1-associated regulation in neurons vs. astrocytes. TAAR1 function appears to be critical for proper neuronal function (20–22, 88), while increased activity in astrocytes may be detrimental to brain health (27, 41, 89). Targeting TAAR1 in the CNS in a cell specific manner may be difficult; however, these findings open the door for personalized medical interventions for these disorders.

**Figure 8:**
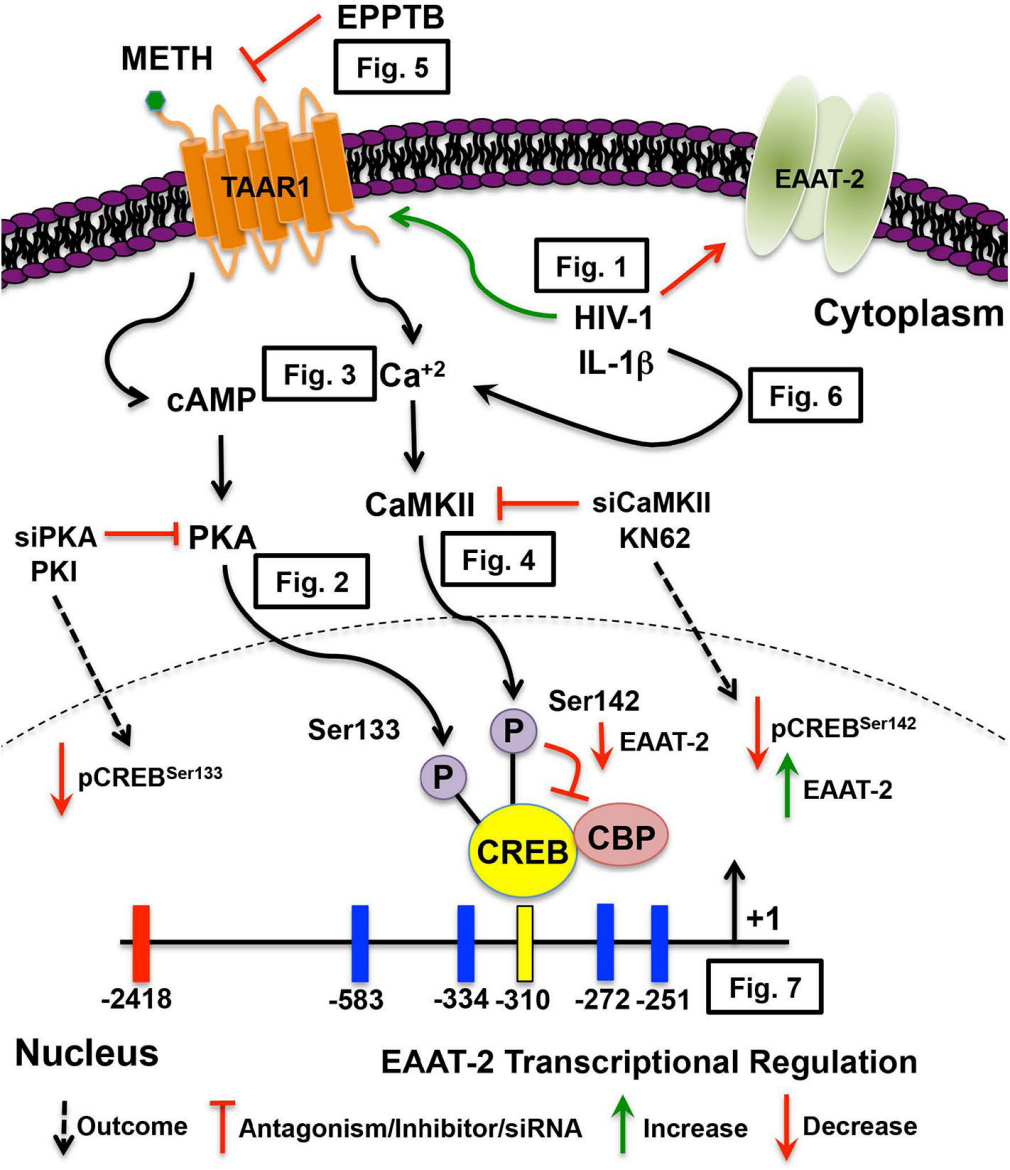
METH-induced TAAR1 signaling regulates astrocyte EAAT-2. METH likely activates different signaling pathways in astrocytes that are exacerbated by HIV relevant inflammatory cytokine, IL-1β and/or HIV-1. Intracellular signal transduction pathways lead to CREB phosphorylation, thereby differentially regulating EAAT-2. We have previously demonstrated METH-induced activation of astrocyte TAAR1 increases intracellular cAMP and regulates EAAT-2. In this manuscript, TAAR1 upregulation and increased activity negatively correlate to astrocyte EAAT-2 and impair glutamate clearance capabilities as demonstrated by (Figure 1). Investigation of signal transduction pathways revealed METH-induced TAAR1 activation leads to cAMP/PKA/pCREB^Ser133^ **(Figure 2)** and [Ca^+2^]_i_/CaMKII/pCREB^Ser142^ (Figures 3, 4). As METH activates TAAR1, antagonism with TAAR1 selective antagonist, EPPTB, prevents METH-induced increases of intracellular cAMP and [Ca^+2^]_i_, blocking METH-induced phosphorylation of CREB at both serine 133 and 142 (Figure 5). Additionally, exogenous treatment of IL-1β and HIV-1 dually activate cAMP/PKA/pCREB^Ser133^ and [Ca^+2^]_i_/CaMKII/pCREB^Ser142^ suggesting similar mechanisms mediating EAAT-2 downregulation (Figure 6). Extrinsic regulation of signaling factors including PKA and CaMKII not only reduce activation of subsequent signaling but also regulate METH-mediated decreases in astrocyte EAAT-2 (Figure 7). Together, our data suggest that pCREB^Ser142^ acts as a dominant repressor of CREB transcriptional activation. Therefore, therapeutically targeting and inhibiting the [Ca^+2^]_i_/CaMKII/pCREB^Ser142^ signal transduction pathways have broader implications in the context of METH abuse and neuroinflammation.

In this study we investigated the duality of METH-induced signaling pathways leading to EAAT-2 transcriptional repression, thereby exacerbating HIV-1-induced decreases in EAAT-2. Figure 8 illustrates signal transduction pathways mediated via TAAR1, METH, HIV-1, and IL-1β in astrocyte EAAT-2 downregulation. We propose that triggering the preferential activation of cAMP/PKA/pCREB^Ser133^ while inhibiting [Ca^+2^]_i_/CaMKII/pCREB^Ser142^ signaling would alleviate excitotoxic insult via upregulation of astrocyte EAAT-2 transcription. Additionally, counteracting IL-1β and HIV-1 induced upregulation of TAAR1 would reduce METH effects in astrocytes. Transcriptional regulation of astrocyte TAAR1 via NF-κB warrants further investigation. In addition, mutation of the four NF-κB sites within the EAAT-2 promoter will elucidate on the correlation identified between TAAR1 and EAAT-2 levels and function following IL-1β treatment. Altogether, our studies shed light on tripartite signaling of PKA, CaMKII, and NF-κB involved in TAAR1 and EAAT-2 regulation, METH abuse, and HAND.

Taken together, we have identified crucial mechanistic pathways involved in METH-induced astrocyte neurotoxicity in the context of HAND. Our data provide strong evidence to support the notion that manipulation of signal transduction pathways to favor cAMP/PKA/pCREB^Ser133^ and to abolish [Ca^+2^]_i_/CaMKII/pCREB^Ser142^ is a promising strategy for restoring astrocyte EAAT-2 function in the context of METH abuse and HAND.

## Data Availability Statement

The datasets presented in this study can be found in online repositories. The names of the repository/repositories and accession number(s) can be found in the article/supplementary material.

## Ethics Statement

The studies involving human participants were reviewed and approved by North Texas Regional IRB. Written informed consent for participation was not required for this study in accordance with the national legislation and the institutional requirements.

## Author Contributions

AG, IC, and KB conceived, designed and coordinated the study, and wrote the manuscript. All authors reviewed the results and approved the final version of the manuscript.

## Conflict of Interest

The authors declare that the research was conducted in the absence of any commercial or financial relationships that could be construed as a potential conflict of interest.

## Abbreviations

[Ca^+2^]_I_: intracellular calcium concentrations
CaMKII: calcium/calmodulin kinase II
cAMP: cyclic adenosine monophosphate
CBP: CREB binding protein
CNS: central nervous system
CREB: cAMP responsive element binding protein
EAAT-2: excitatory amino acid transporter-2
EPPTB: benzamide N-(3-ethoxyphenyl)-4-(1-pyrrdidinyl)-3-(trifluoromethyl)
GAPDH: glyceraldehyde 3-phosphate dehydrogenase
GCaMP6s: pGP-CMV-GCaMP6s plasmid
GFAP: glial fibrillary acidic protein
HAND: HIV-associated neurocognitive disorders
HIV-1: human immunodeficiency virus-1
IL-1β: interleukin 1 beta
METH: methamphetamine
NF-κB: nuclear factor kappa light chain enhancer of activated B cells
NMDA: N-methyl-D-aspartate receptor
pCREB^Ser133^: CREB phosphorylated at serine 133
pCREB^Ser142^: CREB phosphorylated at serine 142
PKA: protein kinase A
TAAR1: trace amine associated receptor 1.
